# Islet organoids: a new hope for islet transplantation in diabetes

**DOI:** 10.3389/fimmu.2024.1540209

**Published:** 2025-01-21

**Authors:** Xuemei Yu, Chengkong Liu, Zheng Kuang, Siyuan Song, Limin Tian, Yi Wang

**Affiliations:** ^1^ Department of Clinical Nutrition, Sichuan Provincial People’s Hospital, University of Electronic Science and Technology of China, Chengdu, China; ^2^ School of Medicine, University of Electronic Science and Technology of China, Chengdu, China; ^3^ Department of Neuroscience, Baylor College of Medicine, Houston, TX, United States; ^4^ Center for Geriatrics and Endocrinology, Sichuan Provincial People’s Hospital, University of Electronic Science and Technology of China, Chengdu, China; ^5^ Clinical Immunology Translational Medicine Key Laboratory of Sichuan Province, Sichuan Provincial People’s Hospital, University of Electronic Science and Technology of China, Chengdu, China

**Keywords:** islet organoids, diabetes treatment, Procr+ progenitors, chemically induced pluripotent stem cells (CiPSCs), endoderm stem cells (EnSCs), β-cell regeneration, personalized therapy, islet transplantation

## Abstract

Diabetes mellitus, including Type 1 diabetes (T1D) and advanced Type 2 diabetes (T2D), remains a major global health challenge due to the destruction or dysfunction of insulin-producing β-cells. Islet transplantation offers a promising therapeutic strategy. However, it is limited by organ shortage globally and other risk factors. Recent advancements in organoid technology provide transformative solutions for islet regeneration. This review summarized three groundbreaking approaches: islet organoids differentiated from Procr+ pancreatic progenitor cells, chemically induced pluripotent stem cells (CiPSCs), and endoderm stem cells (EnSCs). Procr+ cells exhibit multipotency and potential for *in vivo* activation, offering a scalable and non-invasive strategy for β-cell regeneration. CiPSCs, reprogrammed via small molecules, enable personalized islet therapies with promising clinical outcomes, as demonstrated in T1D patients. EnSC-derived islets (E-islets) offer high differentiation efficiency and therapeutic efficacy, particularly for T2D patients with residual β-cell function. While each approach addresses specific challenges in islet transplantation, further research is needed to optimize scalability, immune compatibility, and long-term functionality. This review highlights the potential of organoid-based technologies to revolutionize diabetes treatment and pave the way for personalized, curative therapies.

## 1 Introduction

Diabetes mellitus, encompassing Type 1 diabetes (T1D) and advanced Type 2 diabetes (T2D), continues to present significant challenges to global health systems (1). T1D arises from autoimmune destruction of insulin-producing β-cells, resulting in absolute insulin deficiency. Conversely, T2D is marked by insulin resistance coupled with a progressive decline in β-cell function (2). Both conditions demand effective and innovative treatments, as existing therapies, such as exogenous insulin administration, often fail to fully replicate the dynamic regulation of blood glucose levels observed in healthy individuals.

Islet transplantation has emerged as a promising approach to achieving glycemic control and mitigating diabetes-associated complications (3). However, its clinical application is restricted by critical challenges, including the scarcity of donor islets, risks of immune rejection, and complications associated with current transplantation procedures (4, 5). These limitations have fueled the search for alternative strategies to generate functional islets *in vitro*.

Organoid technology represents a groundbreaking advancement in regenerative medicine (6). By leveraging Endoderm stem cells (EnSC) (7), Chemically Induced Pluripotent Stem Cells (CiPSC) (8), or protein C receptor positive (Procr+) progenitor cells (9), this approach allows for the generation of functional islet organoids capable of insulin secretion. These organoids provide an abundant and scalable source of β-like cells, offering hope for personalized and potentially curative diabetes therapies. Most organoid-related studies are focused on tumor organoid and drug screening (10). Meanwhile, there are some studies on solid organ organoids, such as islets, liver (11), etc. This review summarizes three pivotal studies that explore the use of pancreatic progenitor cells (Procr+ cells), chemically induced pluripotent stem cells (CiPSCs), and endoderm stem cells (EnSCs) in the development of pancreatic organoids. This review centers on their clinical relevance, benefits, challenges, and future directions.

## 2 Organoids from pancreatic progenitor cells

Pancreatic progenitor cells, specifically Procr+ (protein C receptor-positive) cells, have emerged as a novel multipotent cell population within the adult pancreas. These cells, identified by Wang et al. (9), demonstrate the capacity to differentiate into all major endocrine cell types found in the islets, including insulin-producing β-cells, glucagon-secreting α-cells, and somatostatin-producing δ-cells. This discovery highlights the potential of Procr+ cells as a renewable source for islet regeneration and offers a promising alternative to address the challenges of donor islet scarcity.

Procr+ progenitor cells were initially identified in the pancreatic islet niche through lineage-tracing experiments, which confirmed their ability to contribute to all four endocrine cell types of the pancreas. Procr (protein C receptor) serves as a specific marker, distinguishing this rare population from other islet cells. Molecular profiling of Procr+ cells revealed a progenitor-like transcriptional signature, distinct from mature endocrine cells. This unique profile underscores their potential for both regenerative applications and fundamental studies into islet biology. The study further employed single-cell RNA sequencing (scRNA-seq) to elucidate the molecular pathways regulating Procr+ cell differentiation and maintenance. Pathway analyses identified key regulators that could be targeted to enhance β-cell generation efficiency. Such insights provide a framework for optimizing the therapeutic application of Procr+ cells.

A significant advancement in this study was the establishment of a three-dimensional (3D) culture system to expand Procr+ progenitor cells and generate pancreatic islet organoids. These organoids retained their multipotent capacity, enabling long-term *in vitro* expansion while preserving the ability to differentiate into functional endocrine cells. Importantly, β-cells derived from Procr+ organoids exhibited glucose-stimulated insulin secretion (GSIS), a hallmark of mature β-cell functionality.

The therapeutic relevance of these organoids was demonstrated in diabetic mouse models. Following transplantation under the kidney capsule, Procr+-derived organoids restored normoglycemia, improved glucose tolerance, and provided sustained metabolic stability. Human C-peptide secretion in response to glucose stimulation confirmed the functional maturity of the transplanted β-cells, reinforcing their potential for clinical application.

The identification and utilization of Procr+ progenitor cells offer transformative potential for diabetes treatment. These cells can be expanded *in vitro* to produce a virtually unlimited supply of β-cells, addressing the critical shortage of donor islets and providing a scalable solution for islet replacement therapy. Moreover, the ability to isolate Procr+ cells directly from patients paves the way for personalized, autologous treatments, significantly reducing the risk of immune rejection and eliminating the need for lifelong immunosuppression. Additionally, the prospect of activating Procr+ cells *in vivo* presents an opportunity for endogenous islet regeneration, offering a less invasive alternative to traditional transplantation approaches while harnessing the body’s innate capacity for repair.

However, challenges remain in translating these findings to human systems. While Procr+ progenitors have been identified in mice, their existence and characteristics in human pancreatic tissue are yet to be validated. Confirming their presence and functionality in human islets is a critical step toward clinical applicability.

Before forming into the islet organoid, the researchers from the same group observed that Procr+ cells is critical for ovarian surface epithelium by repairing ovulatory rupture and maintaining homeostasis (12). Two year after their study on the islet organoid, they investigated the molecular mechanism on how the Procr+ cells maintain self-renewal characteristics. As Procr+ cells has been deemed as one of the stem cell surface markers in several tissues, mechanisms studies indicated that it could bind with heat shock protein 90 (HSP90), and subsequently form Src and IGF1R at the cell membrane to maintain the stemness (13). Unfortunately, their group did not further explore the potential clinical application of Procr+ cells-induced islet organoids in either non-human primate (NHP) or clinical trial on diabetes patients. In recent five years, there have been some studies on Procr+ cells from other group. Studies on the microenvironment homeostasis of hematopoiesis revealed that Procr+ endothelial progenitor cells could differentiate to various subtypes of vascular endothelial cells through Notch signaling pathway (14). Another study on the follicle development revealed that Procr-expressing granulosa cells are highly proliferative (15). Procr+ cell could also maintain the stemness of cancer stem cell by activating lipid synthesis (16). However, none of these groups continued their study on the islet organoid-related studies.

Future studies should focus on developing pharmacological or gene-editing tools to stimulate Procr+ cells within the pancreas could provide a breakthrough in non-invasive regenerative therapies. Moreover, ensuring the long-term stability and functional integration of β-cells derived from Procr+ organoids is essential to their therapeutic success.

## 3 Islets from chemically induced pluripotent stem cells

Pluripotent stem cells, including embryonic stem cells (ESCs) and induced pluripotent stem cells (iPSCs), have long been considered viable sources for β-cell generation. However, chemically induced pluripotent stem cells (CiPSCs) offer a safer and more scalable alternative. These cells are reprogrammed using small molecules, bypassing the genomic alterations associated with traditional genetic reprogramming.

In a groundbreaking clinical study, Wang et al. (8) demonstrated the efficacy of CiPSC-derived islets in a T1D patient. The study involved reprogramming adipose-derived mesenchymal stromal cells into CiPSCs, which were subsequently differentiated into islets. By staining and single-cell sequencing, these CiPSCs-islets consist of 60% β cells, 10% α-like cells, 10% δ-like cells, 13% endocrine progenitor cells, and ∼8% enterochromaffin-like cells. Before the clinical trial, these CiPSCs-islets were tested on immunodeficient mice and nonhuman primates, to evaluate its safety. No teratoma was found in major organs such as the heart, liver, and others (17). Moreover, these CiPSCs-islets were also undergoing quality control assays as residual PSCs by digital droplet PCR and *de novo* genomic aberrations by whole genome/exome sequencing (WGS/WES). These assays confirmed the efficacy and safety of CiPSCs.

These CiPSCs-islets were transplanted beneath the abdominal anterior rectus sheath, a novel transplantation site that offered several advantages, including reduced inflammation, minimized procedural risks, and easier monitoring of graft function. The total amount of islet transplanted is 1,488,283 IEQ (19,843 islet equivalent (IEQ)/kg).

Clinical outcomes were remarkable: the patient achieved insulin independence within 75 days post-transplantation and maintained glycemic stability for over a year. Glycated hemoglobin (HbA1c) levels improved from 7.57% to below 5.7%, and continuous glucose monitoring revealed a significant increase in time-in-range (TIR) metrics, exceeding 98%. Importantly, no adverse events, such as teratoma formation or graft-related complications (including pain, nausea, vomiting, upper respiratory tract infection, tumor biomarkers), were observed during the follow-up period.

While this study underscores the therapeutic potential of CiPSCs-islets, challenges remain. The requirement for immunosuppression limits its applicability to broader patient populations. Additionally, the long-term durability and scalability of CiPSCs-islets require further validation. Ongoing research should also focus on improving reprogramming efficiency and exploring immune-evasive strategies to enhance graft acceptance in immunocompetent individuals.

## 4 Islet tissue from endoderm stem cells

Endoderm stem cells (EnSCs) represent another promising source for generating islet tissue. As developmentally closer to pancreatic lineages, EnSCs offer the advantage of high differentiation efficiency and minimal tumorigenic risk. Wu et al. (7) conducted a pioneering clinical trial demonstrating the potential of EnSC-islet tissue (E-islets) in a T2D patient. Before the clinical trial, the efficacy of E-islets was tested on Streptozotocin (STZ)-induced diabetic mice and monkeys. In the immunodeficient mice, no tumor formation was observed, and the patient’s autologous E-islets were tolerated by his immune system.

The study involved isolating EnSCs from the patient, followed by differentiation into endocrine progenitor cells and subsequent maturation into E-islets. 1.2 million IEQs of E-islets were transplanted into the hepatic portal vein, enabling direct integration with the patient’s circulatory system. Clinical monitoring over 116 weeks revealed substantial improvements in glycemic control. The patient’s TIR increased to 99% by Week 32, and HbA1c levels decreased from 6.6% to 4.6%. Exogenous insulin therapy was discontinued by Week 11, and oral antidiabetic medications were tapered off within a year. No adverse events, including tumor formation or graft-related complications, were reported.

EnSC-islet tissue hold significant potential for T2D patients with residual β-cell function. However, their application in T1D patients with complete β-cell loss poses additional challenges. Furthermore, the scalability of this approach for broader clinical use and the development of “universal islets” to overcome immunological barriers remain critical areas for future investigation.

The three organoid/islets/islet tissue-based approaches—Procr+ progenitors, CiPSCs, and EnSCs—offer distinct advantages and face unique challenges. A comparative analysis is summarized in **Table 1**.

**Table 1:** The comparison of three main protocols for the generation of islet organoids.

Method	Key Steps	Transplantation Site	Outcome	Challenges	Reference
**Procr+ Pancreatic Progenitors**	Activation of multipotency ↓ 3D organoid expansion ↓ Differentiation into endocrine cells	**Preclinical study:** Mouse Models: Kidney capsule	•Restored normoglycemia. •Improved glucose tolerance.	•No human Procr+ progenitor analog identified. •Requires in vivo activation strategies (e.g., pharmacological or gene-editing tools).	(9)
						**Chemically Induced Pluripotent Stem Cells (CiPSCs)**	Reprogramming adipose mesenchymal cells with small molecules ↓ Generation of CiPSCs ↓ Differentiation into islet-like organoids	**Preclinical study:** Mouse & Monkey Models: Kidney capsule	•Restored normoglycemia. •Improved glucose tolerance •No teratomas	•Immunosuppression required. •Long-term graft stability and scalability require further validation.	(15)
**Clinical trial:** T1D Clinical Trial: Abdominal rectus sheath	•Achieved insulin independence (within 75 days). •HbA1c normalized (<5.7%).										
**Endoderm Stem Cells (EnSCs)**	Patient-derived EnSCs ↓ Endocrine progenitors ↓ Mature E-islets ↓ Transplantation of E-islets for T2D application.	**Preclinical study:** Mouse & Monkey Models: Kidney capsule	•Restored normoglycemia. •Improved glucose tolerance •No teratomas	•Limited to T2D patients with residual β-cell function. •Scalability and universal application require further research.	(17)
		**Clinical trial:** Hepatic portal vein	•Improved Time-in-Range (TIR): 99% at Week 32. •Reduced HbA1c: 6.6% → 4.6%. •Insulin independence achieved by Week 11.		

## 5 Conclusion

Organoid-based technologies herald a transformative era in diabetes treatment, addressing critical limitations of traditional therapies. By leveraging the unique properties of Procr+ progenitors, CiPSCs, and EnSCs, these approaches provide scalable, safe, and potentially curative solutions for both T1D and T2D. Each strategy offers tailored advantages for specific patient populations, ranging from personalized therapy to non-invasive regeneration.

However, significant challenges remain. Future research must focus on identifying human counterparts to Procr+ cells, optimizing immune-evasive strategies, and conducting large-scale clinical trials to validate long-term safety and efficacy. Developing universal “off-the-shelf” islet products could also expand accessibility and reduce reliance on personalized approaches.

## 6 Future perspectives

The continued evolution of organoid technology, combined with advances in gene editing and immune-engineering, holds the potential to revolutionize diabetes care. Collaborative efforts across disciplines will be essential to refine organoid generation protocols, enhance transplantation strategies, and ultimately deliver sustainable, life-changing therapies for millions of diabetes patients worldwide.

## References

[1] Zimmet PZ Magliano DJ Herman WH Shaw JE. Diabetes: a 21st century challenge. Lancet Diabetes Endocrinol. (2014) 2:56–64. doi: 10.1016/S2213-8587(13)70112-8 24622669

[2] DeFronzo RA Ferrannini E Groop L Henry RR Herman WH Holst JJ. Type 2 diabetes mellitus. Nat Rev Dis Primers. (2015) 1:15019. doi: 10.1038/nrdp.2015.19 27189025

[3] Forbes S Kay TWH. Islet transplantation in kidney transplant recipients with type 1 diabetes. Lancet Diabetes Endocrinol. (2024) 12:683–5. doi: 10.1016/S2213-8587(24)00274-2 39250919

[4] Mantovani MDC Gabanyi I Pantanali CA Santos VR Correa-Giannella MLC Sogayar MC. Islet transplantation: overcoming the organ shortage. Diabetol Metab Syndr. (2023) 15:144. doi: 10.1186/s13098-023-01089-8 37391848 PMC10314390

[5] Ricordi C Strom TB. Clinical islet transplantation: advances and immunological challenges. Nat Rev Immunol. (2004) 4:259–68. doi: 10.1038/nri1332 15057784

[6] Rossi G Manfrin A Lutolf MP. Progress and potential in organoid research. Nat Rev Genet. (2018) 19:671–87. doi: 10.1038/s41576-018-0051-9 30228295

[7] Wu J Li T Guo M Ji J Meng X Fu T. Treating a type 2 diabetic patient with impaired pancreatic islet function by personalized endoderm stem cell-derived islet tissue. Cell Discovery. (2024) 10:45. doi: 10.1038/s41421-024-00662-3 38684699 PMC11058776

[8] Wang S Du Y Zhang B Meng G Liu Z Liew SY. Transplantation of chemically induced pluripotent stem-cell-derived islets under abdominal anterior rectus sheath in a type 1 diabetes patient. Cell. (2024) 187:6152–64 e18. doi: 10.1016/j.cell.2024.09.004 39326417

[9] Wang D Wang J Bai L Pan H Feng H Clevers H. Long-term expansion of pancreatic islet organoids from resident procr(+) progenitors. Cell. (2020) 180:1198–211 e19. doi: 10.1016/j.cell.2020.02.048 32200801

[10] Veninga V Voest EE. Tumor organoids: Opportunities and challenges to guide precision medicine. Cancer Cell. (2021) 39:1190–201. doi: 10.1016/j.ccell.2021.07.020 34416168

[11] Lee J Gil D Park H Lee Y Mun SJ Shin Y. A multicellular liver organoid model for investigating hepatitis C virus infection and nonalcoholic fatty liver disease progression. Hepatology. (2024) 80:186–201. doi: 10.1097/HEP.0000000000000683 37976400

[12] Wang J Wang D Chu K Li W Zeng YA. Procr-expressing progenitor cells are responsible for murine ovulatory rupture repair of ovarian surface epithelium. Nat Commun. (2019) 10:4966. doi: 10.1038/s41467-019-12935-7 31672973 PMC6823351

[13] Liu C Lin C Wang D Wang J Tao Y Li Y. Procr functions as a signaling receptor and is essential for the maintenance and self-renewal of mammary stem cells. Cell Rep. (2022) 38:110548. doi: 10.1016/j.celrep.2022.110548 35320720

[14] Xu C Lv X Lv Y Yang S Chen Q Cheng T. Procr+ Endothelial progenitor cells modulate adult hematopoiesis and microenvironment homeostasis via notch signaling. Blood. (2024) 144:562. doi: 10.1182/blood-2024-202263

[15] Wang J Chu K Wang Y Li J Fu J Zeng YA. Procr-expressing granulosa cells are highly proliferative and are important for follicle development. iScience. (2021) 24:102065. doi: 10.1016/j.isci.2021.102065 33644709 PMC7889980

[16] Zhang P He Q Wang Y Zhou G Chen Y Tang L. Protein C receptor maintains cancer stem cell properties via activating lipid synthesis in nasopharyngeal carcinoma. Signal Transduct Target Ther. (2022) 7:46. doi: 10.1038/s41392-021-00866-z 35169126 PMC8847456

[17] Liang Z Sun D Lu S Lei Z Wang S Luo Z. Implantation underneath the abdominal anterior rectus sheath enables effective and functional engraftment of stem-cell-derived islets. Nat Metab. (2023) 5:29–40. doi: 10.1038/s42255-022-00713-7 36624157

